# A model for generating circadian rhythm by coupling ultradian oscillators

**DOI:** 10.1186/1742-4682-3-12

**Published:** 2006-02-23

**Authors:** Verner Paetkau, Roderick Edwards, Reinhard Illner

**Affiliations:** 1 Department of Biochemistry and MicrobiologyUniversity of Victoria Victoria, British Columbia, Canada; 2 Department of Mathematics and Statistics University of VictoriaVictoria, British Columbia, Canada

## Abstract

**Background:**

Organisms ranging from humans to cyanobacteria undergo circadian rhythm, that is, variations in behavior that cycle over a period about 24 hours in length. A fundamental property of circadian rhythm is that it is free-running, and continues with a period close to 24 hours in the absence of light cycles or other external cues. Regulatory networks involving feedback inhibition and feedforward stimulation of mRNA transcription and translation are thought to be critical for many circadian mechanisms, and genes coding for essential components of circadian rhythm have been identified in several organisms. However, it is not clear how such components are organized to generate a circadian oscillation.

**Results:**

We propose a model in which two independent transcriptional-translational oscillators with periods much shorter than 24 hours are coupled to drive a forced oscillator that has a circadian period, using mechanisms and parameters of conventional molecular biology. Furthermore, the resulting circadian oscillator can be entrained by an external light-dark cycle through known mechanisms. We rationalize the mathematical basis for the observed behavior of the model, and show that the behavior is not dependent on the details of the component ultradian oscillators but occurs even if quite generalized basic oscillators are used.

**Conclusion:**

We conclude that coupled, independent, transcriptional-translational oscillators with relatively short periods can be the basis for circadian oscillators. The resulting circadian oscillator can be entrained by 24-hour light-dark cycles, and the model suggests a mechanism for its evolution.

## Background

One of the central puzzles regarding circadian rhythm is the nature of the cellular machinery responsible for it [1]. Although numerous genes required for circadian rhythm have been identified in *Drosophila *[2,3] and other organisms, including cyanobacteria [4], the actual mechanism whereby their products give rise to stable 24-hour oscillations is not established in most cases. Two interesting features have recently been highlighted in reviews: first, that different organisms have different as well as (sometimes) homologous components in their circadian oscillators; and second, that even when components are homologous between organisms, they may function in different ways [1,5,6]. Thus, there may be principles of organization and function that transcend the specific components involved.

Most circadian oscillators are thought to exist within single cells [1,7,8]. Consistent with this, transcriptional-translational feedback circuits ("transcriptional-translational oscillators", or TTOs) are central to most models [1,4], although not to all [9,10]. In a remarkable recent study, a circadian oscillator has been reconstituted that contains only three cyanobacteria-derived proteins in homogeneous solution [11], but this so far appears exceptional.

Ultradian oscillators, i.e. oscillators with periods much less than 24 hours, are ubiquitous in biology, and several authors have suggested that at least some circadian oscillators comprise coupled ultradian ones [12,13]. Examples of ultradian oscillations include 3-hour cycles of expression of the mammalian p53 protein [14], 2-hour periodicity in the expression of the Notch effector Hes1 in cultured cells [15], a 1.5–3 hour periodicity in the expression of NF-κB signaling molecule in mouse cells in culture [16], and a 40-minute cycle in general transcriptional activity in yeast [17]. These systems are members of a broader collection of ultradian oscillators, examples of which include [18] oxygen consumption and other metabolic processes in *Acanthamoeba castellanii*, which have a period of 69 minutes, respiration in *Dictyostelium*, with a period of 60 minutes, and energy metabolism in yeast, which shows the same 40-minute period as much of its transcriptional activity [7].

The idea of generating slow rhythms from relatively fast biochemical processes goes back at least to 1960 [19]. The presence of 'beats' was noted in several experimental studies [20,21], and has been suggested as a mechanism for producing circadian oscillations. It was also suggested that, at least in multicellular organisms, weak coupling of ultradian oscillators between cells can produce circadian oscillations [12,13,22-24]. The 'beats' mechanism has been largely ignored because of a number of critical arguments (cf. [24]), but most of the criticisms predated the gene regulatory model of circadian oscillations. In this paper we invoke a phenomenon somewhat related to 'beats' as a way of using ultradian cycles to generate circadian ones within a single cell.

More recently, several models for TTO circadian oscillations have been developed that do not depend on ultradian oscillators as components. One of these [25,26] comprises two genes, one producing a transcriptional activator and the other a repressor, each of which affects both itself and the other gene. In addition, the activator and repressor proteins combine into a dimer, which inactivates them both. Another model for a mammalian TTO, comprising interacting positive and negative regulatory loops, involves the products of *Per*, *Cry*, *Bmal1*, *Clock *and *Rev*-*Erb*α genes, and also produces circadian oscillations and entrainment to light-dark cycles [27]. A similar model for the circadian oscillator in *Drosophila *involves a complex of the products of *Per *and *Tim *[28]. These examples involve closely-interlinked TTO components. Interestingly, it was the circadian clock in *Drosophila *that prompted the modeling of circadian rhythms as coupled ultradian ones [12], and this proposal was based partly on data showing ultradian peaks in the power spectrum.

A model proposing that circadian oscillators have evolved from pre-existing ultradian ones involves five ultradian oscillators arranged in a loop [29]. We describe here a different kind of coupled ultradian model, in which two independent ultradian TTOs drive a third oscillator by the combination of their protein products. In this model, the frequency of the output is related to the difference in frequencies between the two independent primary oscillators. Neither the early papers suggesting 'beats' as a mechanism [20,21] nor the proposed mathematical models involving populations of ultradian oscillators [12,13,24] include mechanistic or molecular details. In this paper, we demonstrate that realistic mechanisms and parameters taken from molecular biology can produce a circadian oscillator using ultradian component TTOs. The model also suggests a mechanism for its evolution.

## Results

### Overview of the model

Our model contains two coupled ultradian TTOs that generate circadian oscillations within a single cell. It does not involve transport across cellular membranes or molecular modifications such as methylation. The primary feature of the model is that linking the output of independent ultradian TTOs of slightly different frequencies generates a circadian rhythm.

**Figure 1:**
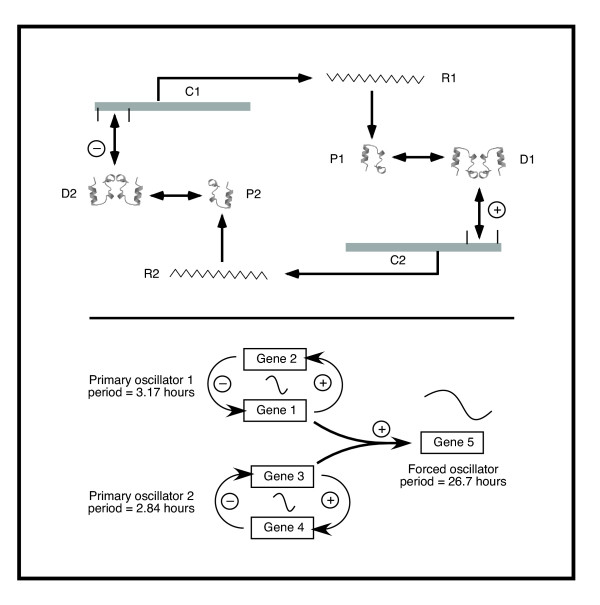
**Model of a 5-gene circadian oscillator. **The components of the first of the primary oscillators are illustrated in the top half of the figure. C1, C2 – the genes coding for R1 and R2; R1, R2, the mRNAs encoding the proteins P1 and P2; P1, P2, the protein products, which undergo association to dimers D1 and D2, respectively. D1 stimulates the transcription of C2 by binding to its regulatory region, and D2 inhibits the transcription of C1 by binding its regulatory region. The decays of mRNAs and proteins are not shown. The overall model is shown in the lower half of the figure. It comprises two independent, ultradian, primary oscillators (genes 1+2 and 3+4, respectively), in which the homodimeric protein product of gene 1 positively regulates the transcription of gene 2, and a homodimer of protein 2 inhibits transcription of gene 1. Genes 3 and 4 are similarly related. The two primary oscillators differ slightly in their respective periods. The protein products of genes 1 and 3 form heterodimers that regulate the transcription of the fifth gene (the forced oscillator). In the present model, and using the parameters given (Figure 2 legend), the periods of the primary oscillators are around 3 hours, while the period of the fifth gene in the absence of light-dark coupling is just over 26 hours.

The model is outlined in Figure 1. It is based on two self-sustaining TTOs ("primary oscillators") with different ultradian frequencies, each producing transcription-regulating proteins that form homodimers. Examples of homodimeric transcriptional regulators (complexes of 2 identical protein molecules), and heterodimeric ones (dimers containing 2 different protein molecules) are well known [30], and some have been identified as parts of known cellular oscillators [16,31,32], including other models of circadian oscillators [28]. Each of the primary oscillators in the model is regulated by its own homodimeric protein products. A heterodimeric complex containing one protein molecule from each of the two primary oscillators activates transcription of a forced oscillator, giving it (the forced oscillator) a behavior that has a complex relationship with the frequencies of the primary oscillators. By the nature of the coupling between the protein products of the primary oscillators, the driven oscillator (gene 5 in Figure 1) can have a period much longer than either of the two primary oscillators.

A variety of feedback-inhibited gene regulation models can be constructed using known molecular interactions, including (among others) transcriptional repression and induction, phosphorylation of control proteins and inhibition of inducers by complex formation and promoter methylation [3,33-35]. We have used a fairly simple model for the primary oscillators, since their nature is not critical to the principle of the model (although their ability to cooperate is). Each primary oscillator comprises two genes, and the protein products of each gene form homodimers that regulate the other. Gene 1 protein homodimers stimulate transcription of gene 2, and gene 2 protein homodimers repress transcription of gene 1. The same relationships occur in genes 3 and 4, which comprise the second primary oscillator. The two primary oscillators have slightly different periods of around 3 hours, similar to a number of known transcriptional oscillators [14,16,36,37]; the slight difference is critical to the model.

Coupling between the primary oscillators is achieved through the formation of heterodimeric complexes of the protein products of genes 1 and 3. These heterodimers bind to the fifth gene and stimulate its transcription, forcing it to undergo oscillations of which the period is a function of the frequency difference between the two primary oscillators. Properly chosen, the slight difference in frequencies of the primary oscillators induces a rise and fall in the concentration of the heterodimeric product that generates circadian oscillation of the expression of gene 5.

### The first primary oscillator

Each primary oscillator consists of two genes that are transcribed and translated, and the protein products generated then form homodimers as described, with the homodimeric protein product of the second gene binding to the first gene and inhibiting its transcription, and the homodimeric protein product of the first gene binding to the second gene and inducing its transcription (Figure 1). Translation is assumed to be proportional to the level of mRNA. All interactions are described by kinetic equations.

The first primary oscillator is described by the following differential equations:

    (1) dC_1_/dt = k_11_(DNA-C_1_)D_2 _- k_12_C_1_

    (2) dR_1_/dt = k_13_(DNA-C_1_) + L_1 _- k_14_R_1_

    (3) dP_1_/dt = k_15_R_1 _- k_16_P_1 _- 2k_17_P_1_^2 ^+ 2k_18_D_1 _- k_61_P_1_P_3 _+ k_62_D_13_

    (4) dD_1_/dt = k_17_P_1_^2 ^- k_18_D_1 _- k_21_(DNA-C_2_)D_1 _+ k_22_C_2_

    (5) dC_2_/dt = k_21_(DNA-C_2_)D_1 _- k_22_C_2_

    (6) dR_2_/dt = k_23_C_2 _+ L_2 _- k_14_R_2_

    (7) dP_2_/dt = k_25_R_2 _- k_16_P_2 _- 2k_17_P_2_^2 ^+ 2k_18_D_2 _- k_29_LP_2_

    (8) dD_2_/dt = k_17_P_2_^2 ^- k_18_D_2 _- k_11_(DNA - C_1_)D_2 _+ k_12_C_1_

where the first 4 equations describe the behavior of gene 1 and its products, and equations 5–8 describe gene 2. In these equations, R_1_, P_1_, and D_1 _respectively represent mRNA, protein and the protein homodimer of gene 1, and R_2_, P_2 _and D_2 _are the corresponding products of gene 2. C_1 _represents gene 1 that has formed a complex with the repressor protein dimer D_2_, and C_2 _the complex between gene 2 and D_1_. "DNA" is the total concentration of each gene, taken to be 1 × 10^-9 ^M. Binding of D_2 _to gene 1 (Equation 1) represses its transcription, so that the rate of change of R_1 _(equation 2) is proportional to the amount of unbound gene 1, plus L_1_, ("leakage", which is transcription in the presence of saturating D_2_) and degradation. For simplicity, degradation of RNA and protein are taken to be first order. Although such reactions are undoubtedly carried out by enzymes, i.e. saturable catalysts, it is unlikely that the variations in macromolecular species seen here would change the overall cellular concentrations of mRNA and protein, and thus first-order processes suffice. The rate of change in P_1 _(equation 3) is a function of its translation from R_1_, its degradation, the formation and dissociation of homodimer D_1 _(equation 4), and formation and dissociation of heterodimer D_13 _(equation 17, below). Finally, the change in the concentration of the homodimer D_1 _(equation 4) is the result of its formation by the dimerization of P_1_, its own dissociation, and its binding to and dissociation from gene 2.

Equations 5–8 describe the behavior related to gene 2, which differs from gene 1 in two ways. First, its transcription is positively controlled (induced) by the binding of D_1_, and is thus proportional to the level of the complex C_2_. Secondly, the protein product of gene 2, P_2_, is degraded by a light-dependent mechanism through a coupling constant k_29_. Such an activity has recently been ascribed to Cryptochrome, the blue light-sensitive protein that causes the rapid proteolysis of the Tim protein of the *Drosophila *circadian oscillator [38]. The variable "L" (light) in equation 7 has a value between 0 and 1, representing dark and full daylight, respectively. Behavior of the system with L = 0 (that is, in continuous darkness) or in continuous light (L = 1) is used to determine circadian behavior (the function describing L is given in the legend to Figure 4). The other components of the gene 2 system (equations 5–8) are parallel to those of gene 1 (equations 1–4).

Some of the parallel parameters for the two genes in the first primary oscillator were given the same values. These included the first order constant for mRNA degradation, k_14_, which corresponds to an 8-minute half-life (the choices of parameters are rationalized in the Discussion). The parameter for protein degradation, k_16_, was given a value corresponding to a 10-minute half-life, and the association and dissociation rates of the protein homodimers (k_17 _and k_18_, respectively) were the same for the two genes. The "leakiness" of each gene (the value assigned to transcription in either the fully repressed or uninduced states) was set to 0.1% of the maximum rate of transcription for every gene in the system. As a result of these simplifications, each primary oscillator contains 14 different parameters (including the concentration of DNA).

The primary oscillator represented by these equations contains an odd number (namely 1) of negative feedback arms, as required to produce oscillation [36,39], and has a degree of association of protein elements (cooperativity) of 2 (i.e. the proteins form dimers).

### The second oscillator

Since the exact nature of the primary oscillators is not critical, as long as they reflect realistic and plausible biochemical mechanisms, the second oscillator is taken to have exactly the same structure as the first, with the critical difference that it has a slightly shorter period. To achieve this most simply, we have multiplied all of the rate equations for the first primary oscillator by a factor slightly greater than 1 (δ = 1.125) in describing the second, thereby giving the second primary oscillator a period about 12% shorter. In this case, all processes, including e.g. the rates of decay of mRNA and protein are scaled. Equations 9–16 describe the second primary oscillator.

    (9) dC_3_/dt = δ(k_11_(DNA-C_3_)D_4 _- k_12_C_3_)

    (10) dR_3_/dt = δ(k_13_(DNA-C_3_) + L_1 _- k_14_R_3_)

    (11) dP_3_/dt = δ(k_15_R_3 _- k_16_P_3 _- 2k_17_P_3_^2 ^+ 2k_18_D_3 _- k_61_P_1_P_3 _+ k_62_D_13_)

    (12) dD_3_/dt = δ(k_17_P_3_^2 ^- k_18_D_3 _- k_21_(DNA-C_4_)D_3 _+ k_22_C_4_)

    (13) dC_4_/dt = δ(k_21_(DNA-C_4_)D_3 _- k_22_C_4_)

    (14) dR_4_/dt = δ(k_23_C_4 _+ L_2 _- k_14_R_4_)

    (15) dP_4_/dt = δ(k_25_R_4 _- k_16_P_4 _- 2k_17_P_4_^2 ^+ 2k_18_D_4 _- k_29_LP_4_)

    (16) dD_4_/dt = δ(k_17_P_4_^2 ^- k_18_D_4 _- k_11_(DNA - C_3_)D_4 _+ k_12_C_3_)

### The forced oscillator

The fifth gene, which is the forced oscillator, is positively regulated by the heterodimer (D_13_) consisting of P_1 _and P_3_. The protein products of genes 1 and 3 form the dimer (equation 17, below), which binds to gene 5 and induces its transcription. The product of this transcription is translated and dimerizes to form D_5_, which controls other cellular functions with a circadian period. The behavior of the fifth gene is given by the following equations, which have the same structure as those used for the primary oscillators:

    (17) dD_13_/dt = k_61_P_1_P_3 _- k_62_D_13 _- k_21_(DNA-C_5_) D_13 _+ k_52_C_5_

    (18) dC_5_/dt = k_21_(DNA-C_5_)D_13 _- k_52_C_5_

    (19) dR_5_/dt = k_53_C_5 _+ L_5 _- k_54_R_5_

    (20) dP_5_/dt = k_55_R_5 _- k_56_P_5 _- 2k_57_P5^2 ^+ k_58_D_5_

    (21) dD_5_/dt = k_57_P_5_^2 ^- k_58_D_5_

As for the primary oscillators, transcriptional "leakage" is included (L_5_).

### Behavior of the model

**Figure 2:**
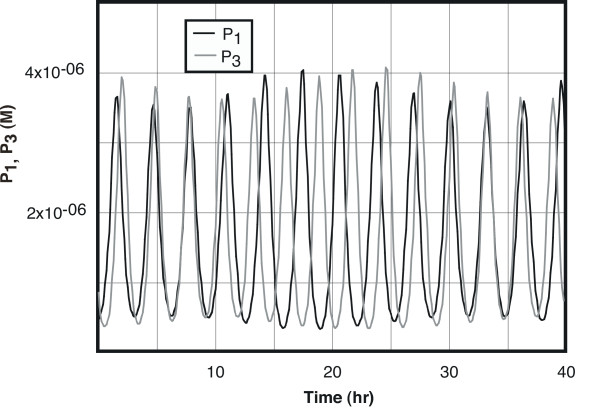
**Behavior of the two primary oscillators. **The molar concentrations of the protein products of the two primary oscillators, P_1 _and P_3_, are shown as a function of time. The data were generated using the system of equations described in the text, with the parameters given below, and in constant darkness. The period over which the relative positions of the two primary oscillators repeat corresponds to the slow circadian frequency seen for the system overall (26.7 hours). Parameters used in the model: k_11 _= 1 × 10^9^/(M • h), k_12 _= 0.3/h, k_13 _= 2000/h, k_14 _= 5.2/h, k_15 _= 500/h, k_16 _= 4.1/h, k_17 _= 5 × 10^5^/(M • h), k_18 _= 15/h, k_21 _= 1.2 × 10^6^/(M • h), k_22 _= 2/h, k_23 _= 600/h, k_25 _= 400/h, k_29 _= 4, k_52 _= 0.7/h, k_53 _= 1500/h, k_54 _= 2.55/h, k_55 _= 8/h, k_56 _= 2/h, k_57 _= 5 × 10^6^/(M • h), k_58 _= 10/h, k_61 _= 2 × 10^5^/(M • h), k_62 _= 2/h, DNA = 1 × 10^-9 ^M, δ = 1.125, L_1 _= 2 × 10^-9^M/h, L_2 _= 6 × 10^-10^M/h, L_5 _= 1.5 × 10^-9^M/h.

Numerical solution of this set of differential equations using the program XPP [40] shows that genes 1, 3 and 5 have periods of 3.17, 2.84, and 26.7 hours, respectively. The behavior of P_1 _and P_3 _is shown in Figure 2. The ratio between their periods is 1.116, not precisely the value of δ, 1.125, because of the slight coupling between P_1 _and P_3 _through the formation of D_13 _and its binding to gene 5. This coupling is reflected in the varying amplitudes of D_1 _and D_3 _seen in Figure 2, a variation that reflects the circadian period of gene 5.

**Figure 3:**
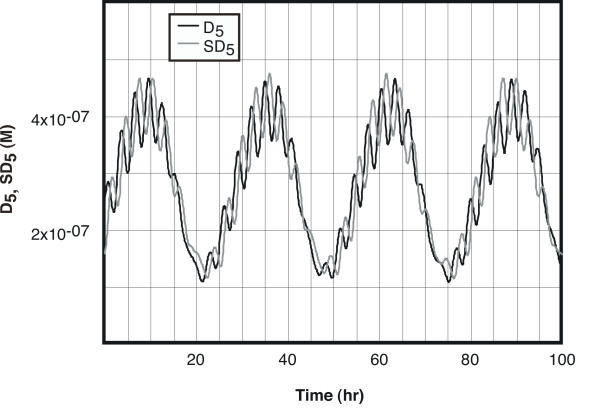
**Behavior of the circadian oscillator under free-running conditions. **The concentration of the homodimeric protein product D_5 _of the forced oscillator (gene 5 in Figure 1) shows both a small, residual short-period fluctuation and a low-frequency oscillation of much higher amplitude, with a period of 26.7 hours in constant darkness. The small, fast oscillations correspond to the average period of the primary oscillators (ca. 3 hours). The lighter (gray) trace represents the behavior of the model in which the primary transcriptional-feedback oscillators of the model are replaced by sine functions (equations 23 and 24). The variable plotted is SD_5_, representing the behavior of D_5 _when it is driven by the sine wave functions.

The behavior of the D_5 _product of gene 5 is shown in Figure 3, which shows a 26.7 hour circadian pattern. On this is superimposed a faster, lower-amplitude pattern that reflects the average period of the primary oscillators.

**Figure 4:**
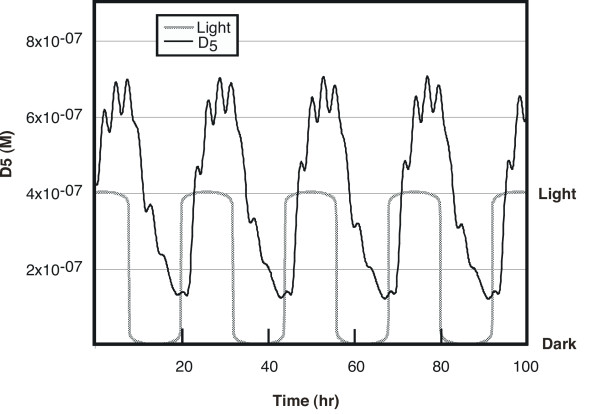
**Entrainment of the circadian oscillator by 24-hour light-dark cycles. **During 12-hour periods of light and dark, the circadian oscillator (D_5_) shows a 24 hour period, owing to a presumed light-activated protease that degrades the products of the driving oscillators. "Light" was represented by a function, L, that varied between 0 (dark) and 1 (light), and was linked to the degradation of the light-sensitive proteins P_2 _and P_4 _(see text) through the coupling constant k_29_. The function used to represent the light/dark cycle was : L = {|sin(2πt/24)|^.05 ^•sign(sin(2πt/24))+1}/2 where t is the time in hours and "sign" is the defined by sign(x) = -1 when x < 0, = 0 when x = 0, and = 1 when x > 0. The effect of light (L = 1) is to decrease the half-lives of proteins P_2 _and P_4 _from 10 minutes to just over 5 minutes.

**Figure 5:**
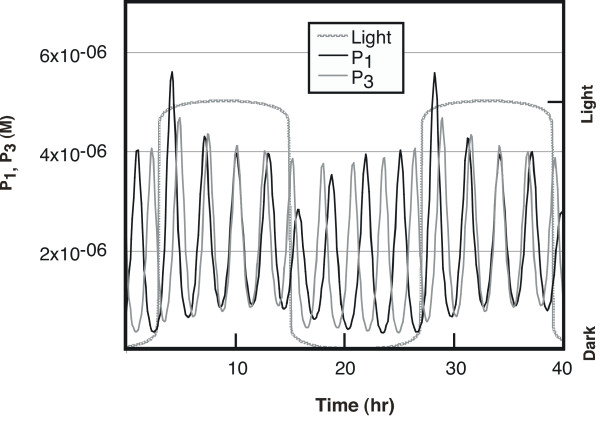
**Effect of light on the primary oscillator products P1 and P3. **In constant darkness (Figure 2), the phases of the two primary oscillators coincide every 26.7 hours, thereby determining the free-running period of the forced oscillator. The effect of 24 hour light/dark periods is to change the period of the two primary oscillators and bring them into phase alignment once each "day", resulting in an entrainment of the circadian oscillator to the 24 period.

When a 24-hour light-dark cycle is imposed, the forced oscillator (gene 5) exhibits a period of 24 hours, owing to the sensitivity of P_2 _and P_4 _to light (Figure 4). This is the result of the two primary oscillators being forced into synchrony in the same part of the light-dark cycle every 24 hours (Figure 5). In constant darkness (Figure 2), the phases of the two primary gene products P_1 _and P_3 _coincide only every 26.7 hours, corresponding to the free-running period of the driven oscillator.

### Mathematical analysis of the system

The basic mathematical patterns in this model are quite simple: the long-period oscillations arise by a double forcing, with two oscillators of slightly different periods driving another system that need not, on its own, oscillate. The crucial feature of the model is that it is the *product *of protein concentrations of the primary oscillators that drives the forced oscillator (equation 17). The effect of using the product of oscillations of similar but non-identical period is to produce a superposition of a fast oscillation and a slow one, at the difference of the two primary frequencies (Figure 3). The integration of this product by the driven system decreases the amplitude of the fast oscillations in comparison to the slow (circadian) ones.

The specific physical nature of the oscillators is not crucial to this model: any similarly-organized system will display the same behavior. A paradigmatic example is

d^2^x/dt^2 ^+ ω^2 ^x = 0,

d^2^y/dt^2 ^+ (ω+ε)^2^y = 0, with ε small relative to ω

dz/dt = -kz + xy,

in which the product of two harmonic oscillations of similar period drives the z variable; or equivalently, using special solutions to the first two equations,

    (22) dz/dt = -kz + sin(ωt) sin((ω+ε)t).

This equation has solutions consisting of a fast, small-amplitude oscillation at frequency (2ω+ε)/(2π) superimposed on a large, slow oscillation at frequency ε /(2π). To see this, note that

2sin(ωt) sin((ω+ε)t) = cos(εt) - cos((2ω+ε)t).

The z variable is thus driven by a long-period oscillation of frequency ε /(2π), and a short-period oscillation of frequency (2ω+ε)/(2π). The higher frequency oscillation has a smaller effect on the amplitude of z because, roughly speaking, z integrates the two driving terms, cos(εt) and -cos((2ω+ε)t, so that they are divided by their frequencies.

This paradigmatic example is not quite the same as the well-known phenomenon of beats arising in linearly coupled oscillators, in which oscillations of similar frequencies are added rather than multiplied. For example,

f(t) = sin(2ωt) + sin(2(ω+ε)t) = 2cos(εt) sin((2ω+ε)t)

displays beats with frequency ε /(2π). However, in our model, the oscillating variables are necessarily strictly positive, whereas a pure sine wave has a mean of zero and the offset to keep it positive does induce beats, as in

f(t) = 2(sin(ωt) + A) (sin((ω+ε)t) + B)

= 2Bsin(ωt) + 2Asin((ω+ε)t) + cos(εt) - cos((2ω+ε)t) + 2AB.

In any case, the faster frequencies still become smaller relative to the slowest frequency after being integrated by the differential equation, especially if A and B are not too large (i.e. if the minimum of the oscillations is close to zero relative to the maximum) and if ω is somewhat larger than the decay rate, 'k' in equation 22, of z.

We compared the behavior of the paradigmatic example with our model by replacing the terms P_1 _and P_3 _in the differential equation for D_13 _(equation 17) by the terms SP_1 _and SP_3_, where

    (23) SP_1 _= A{sin(2πt/Per)/2} + B, and

    (24) SP_3 _= A{sin(2πtΔ /Per)/2} + B

where Per represents the period (chosen to coincide with that of P_1 _in the model, 3.17 hours), and Δ = 1.12 (to give SP_3 _the same frequency as P_3 _in the model). A and B are constants chosen to yield correspondence in behavior to the molecular model. SP_1 _and SP_3 _should be thought of as first order Fourier series approximations of P_1 _and P_3_.

When the sine function oscillators SP_1 _and SP_3 _are used in place of P_1 _and P_3 _to drive the forced oscillator (gene 5), the model produces circadian oscillations (Figure 3) essentially identical to the original model. This indicates that the precise nature of the driving oscillators P_1 _and P_3 _is not important – as long as they have the appropriate frequency relationship, they will generate a forced circadian oscillation in the driven system.

## Discussion

We describe a model that uses transcriptional-translational oscillators of relatively fast (ultradian) frequencies to drive a forced oscillator with a period of approximately 24 hours, i.e. a circadian oscillator. The ultradian oscillators differ in their frequencies, and their products are coupled to force the output oscillator. It is only necessary that the primary oscillators are periodic – sinusoidal oscillators with the same period as the nonlinear transcriptional-translational systems described will drive the forced oscillator in the same way, with a similar fine structure.

The two primary oscillators may differ qualitatively, to avoid having either one alone able to drive the forced oscillator. For example, ultradian cycling of the cellular redox state might alter the effectiveness of a transcription activator with its own independent ultradian rhythm. Indeed, an effect of redox state on a transcription activator of circadian gene expression is known [32]. Because the primary oscillators in our model work in a product fashion, rather than, say, being additive, it is not necessary that their individual products have similar concentration ranges to drive the fifth gene with a circadian period.

It is difficult to relate the parameters in this model to actual values in cells undergoing circadian rhythm, much less to components of circadian oscillators themselves, many of which remain unknown. However, the parameters (Figure 2 legend) are based on plausible values. The most critical values are the degradation rates of mRNA and, to a lesser extent, protein. We have used 8 minutes for the half-life of mRNAs of the primary oscillators, which is similar to several eukaryotic and prokaryotic mRNAs: c-fos mRNA has been reported to have a half-life of 6.6 minutes in NIH 3T3 cells [41] and 9 minutes in human fibroblasts [42], and the average for *E. coli *mRNA has been reported to be 6.8 minutes [43]. The stabilities of individual mRNAs in a cell can differ by orders of magnitude, but the short half-life used in our model is not unrealistic.

The parameter for protein turnover in the model corresponds to a half-life of about 10 minutes. Although the half-life of the average protein in eukaryotic cells is many hours, much faster turnover is found for some proteins, including reported 12 and 18-minute half-lives for rat liver ornithine decarboxylase and δ -aminolevulinate synthetase, respectively [44]. The corresponding value for *Tim*, a component of the *Drosophila *circadian system, is 20 minutes [38]. The half-life of p53 is 16 minutes in a keratinocyte cell line [45], and that of N-myc is 30 minutes [46]. Although prokaryotic proteins typically have half-lives in the order of hours, there are exceptions. For example, 48 proteins of *Caulobacter *turned over much more quickly than the cell cycle time of 120 minutes [47], and the lambda repressor protein in *E. coli *has a half-life of about 60 minutes [48]. More generally in *E. coli*, the majority of proteins turn over slowly, but some are much shorter-lived [49]. In the represillator model of Elowitz and Leibler, the critical proteins were taken to have a half-life of about 10 minutes [36]. In any case, our proposed mechanism is not ultimately dependent on the shorter half-lives we have chosen but on the ratio of the periods of the primary oscillators.

The light-dependent mechanism of phase-resetting in the model is based on the properties of the *Drosophila *Cryptochrome protein, which induces light-activated degradation of Tim protein that is part of that organism's circadian oscillator [38]. In our model, and using the parameters of Figure 2, the half-life of proteins P_2 _and P_4 _are reduced from 10 minutes in the dark to 5.1 in light through the coupling factor k_29_. A more realistic version would probably have the effect of light-driven degradation restricted to only one of the primary oscillators, but we have not pursued this variation.

The output of the model (gene 5 in Figure 1) could provide the kind of circadian timing that would be analogous to the "master regulators" that control the timing of cell cycle events in *Caulobacter *[33]. The evolution of such a circadian system might begin with the development of ultradian TTOs, which themselves have important regulatory value, like that of the NF-κB system [16,35]. The creation of a forced oscillator that responds to the products of two such ultradian oscillators depends on their individual frequencies, the strength of their interactions, and the binding strengths between their products and the transcription control site of the forced oscillator. Thus, the development of a circadian oscillator could occur independently of the functions of the primary oscillators, allowing for the development of a new, beneficial trait (circadian rhythm) without significantly affecting the primary systems. A different model for evolution of circadian systems based on the development of synchronized metabolic pathways has been proposed by Roenneberg and Merrow [29].

Whether any existing circadian oscillators depend on ultradian ones as suggested here or in earlier work [12,13,29] is unproven, but evidence consistent with this model can be seen in power spectral analyses of some circadian systems, including the activity profile of *Drosophila *[12] and the secretion of ghrelin in rats [50], both of which show higher frequency components in addition to the main circadian frequency.

Amongst the arguments that have been brought forward against 'beats' as a mechanism is that coupled oscillators of similar frequencies will undergo mutual entrainment and that the 'beats' will be lost [24]. In our model, oscillators are coupled indirectly and weakly, through the formation of a protein heterodimer. In the case of weak coupling, Pavlidis [24] has argued that the relative phases of the primary oscillators would be random and too much variability of behavior would result. In the model presented here, the primary oscillators do not undergo mutual entrainment, and the output is not dependent on the initial phase relationship between them.

It has also been argued that models based on beats are not robust because small changes in the periods of the primary oscillators lead to large changes in the circadian period [24,51]. In the absence of directly pertinent data, it is difficult to determine whether this is a significant problem. However, the enzymes that carry out biochemical reactions have well defined rate constants, which do not normally change, and thus a shift in frequency would not be expected in such a model. A more fundamental concern is that real reactions are stochastic, and especially under cellular conditions with small numbers of some molecules (for example, the genes involved), this might lead to instability in oscillators of this type. We have therefore also cast the model into stochastic terms, and the results indicate that the system is robust to stochastic fluctuations (work in progress). Finally, a TTO model can provide temperature compensation, since the increase in reaction rates typical of biological processes may be opposed by a decrease in the rate of formation of DNA-binding protein dimers, as has been documented for the leucine zipper transcriptional oscillator GCN4 [30].

The effect of light on the primary oscillators would be selected on the basis of the benefit of making the levels of certain gene products lower or higher in daylight than at night, and could be achieved by a light-sensitive protease such as the Cryptochrome of *Drosophila *[38] before the evolution of the circadian oscillator. Over time, the development of a circadian rhythm might impart larger benefits to the organism. In cyanobacteria, for example, matching of the free-running period to the light-dark cycle time provides a selective advantage [52], which is presumably the basis for its evolution. In *Arabidopsis*, matching between the circadian period and the light-dark cycle results in plants that fix carbon at a higher rate and grow and survive better than those that lack such a match [53].

Cellular oscillators based on metabolic pathways have also been described. Almost 40 years ago, Chance and colleagues described oscillations in glycolytic pathways both in yeast and yeast extracts. In intact cells the oscillations had a high damping factor, but with a judicious choice of long-lasting carbohydrate substrate, enzyme extracts could maintain oscillations for very long times. Furthermore, the basic short period oscillations (in the order of 10 minutes) were sometimes superimposed on slower periodicities that were two or even more times the fundamental frequency [54]. These authors suggested that similar oscillations might be basic regulators of biological clocks. In general, however, oscillators that depend on extracellular substrates are not attractive for this purpose, since the oscillations will fluctuate or even extinguish depending on the levels of those substrates [55]. Mechanisms that are entirely intracellular in terms of substrates and products, such as the one described here, are more likely to provide stable primary oscillators. The only necessary communication with the outside world is through a light-sensitive mechanism to reset the phase of the driven oscillator.

## Conclusion

Independent transcriptional-translational oscillators with relatively short (ultradian) periods can be coupled to generate a circadian oscillator using conventional mechanisms of molecular genetics and reasonable values of parameters describing these mechanisms. The resulting circadian oscillator can be entrained by 24-hour light-dark cycles. The model suggests that evolution of such a circadian oscillator would occur under selective pressure without significantly perturbing the underlying components.

## Methods

Differential equations were solved numerically using the XPPAUT software described by Ermentraut .

## Competing interests

The author(s) declare that they have no competing interests.

## Authors' contributions

VP proposed the original problem of generating circadian oscillations with relatively short-lived molecular processes and wrote the bulk of the paper; RI and RE proposed the coupled oscillator approach, and developed the ordinary differential equation model and the analysis of its behavior. All three authors worked to bring the model to fruition through discussions and analysis of simulations.
